# In vitro hypoxia responsiveness of [^18^F] FDG and [^18^F] FAZA retention: influence of shaking versus stagnant conditions, glass versus polystyrene substrata and cell number down-scaling

**DOI:** 10.1186/s41181-020-00099-5

**Published:** 2020-06-15

**Authors:** Morten Busk, Michael R. Horsman, Jens Overgaard, Steen Jakobsen

**Affiliations:** 1 Experimental Clinical Oncology, Department of Oncology, Aarhus University Hospital (AUH), Palle Juul-Jensens Boulevard 99, 8200 Aarhus, Aarhus N Denmark; 2 Danish Centre for Particle Therapy, Aarhus University Hospital, Aarhus, Denmark; 3 Department of Nuclear Medicine & PET centre, AUH, Aarhus, Denmark

**Keywords:** Hypoxia, Tracer availability, Diffusion, Convection, Cell substrata, In vitro conditions

## Abstract

**Background:**

In vitro experiments using radiolabeled molecules is fundamental for Positron emission tomography (PET) or single photon emission computed tomography (SPECT) tracer development and various metabolic assays, but no consensus on appropriate incubation conditions exists. Specifically, the use of shaking versus non-shaking conditions, cell number to medium volume and the choice of cell plating material may unintentionally influence cellular oxygenation and medium composition. This is problematic when testing the oxygen-dependence of tracers including ^18^F-fluoro-2-deoxyglucose ([^18^F]FDG) and hypoxia-selective 2-nitroimidazoles (e.g., ^18^F-fluoroazomycin-arabinoside, [^18^F]FAZA) or when doing prolonged experiments. The purpose of this study was to assess the influence of various experimental conditions on tracer retention.

**Methods:**

Tumor cells were seeded in a) Glass or standard Polystyrene Petri dishes or as b) discrete droplets in polystyrene Petri dishes or on 9 mm glass coverslips positioned in glass Petri dishes. When confluent, cells were pre-equilibrated for 2 h to 21%, 0.5% or 0% O_2_ and [^18^F] FDG or [^18^F] FAZA was added, followed by cell harvest and analysis of radioactivity 1 h ([^18^F]FDG) or 3 h ([^18^F]FAZA) after. Experiments were conducted with/without orbital shaking.

**Results:**

The influence of hypoxia on tracer retention varied widely among cell lines, but shaking-induced convection did not influence uptake. In contrast, hypoxia-driven [^18^F] FAZA, and to some extent [^18^F] FDG, retention was much lower in cells grown on polyethylene than glass. Scaling-down the number of cells did not compromise accuracy.

**Conclusions:**

Tracer retention was similar under stagnant and forced convection conditions suggesting that the former approach may be appropriate even when accurate control of oxygen and tracer availability is required. In contrast, conventional plasticware should be used with caution when studying tracers and drugs that are metabolized and retained or activated at low O_2_ levels. Downscaling of cell number, by reducing the effective growth area, was feasible, without compromising accuracy.

## Background

Numerous in vitro cell studies involves assessment of radioactive tracers, but there is no consensus on how such experiments ideally should be performed, which in turn may depend on the application. In oncology, there is a special interest in the development of PET tracers that may allow us to quantify metabolic and microenvironmental differences between tumors. For example, the presence of viable hypoxic tumor cells in solid tumors is strongly linked to poor prognosis, and hypoxia driven PET tracer retention has been widely studied as a means to identify patients with hypoxic tumors (Horsman et al. 2012). Focus has mainly been on a) assessing the stimulation of anaerobic glycolysis in hypoxic cells which may principally be determined from [^18^F] FDG retention or b) the retention of hypoxia selective 2-nitroimidazoles which are reduced and retained at low oxygen levels (Busk et al. 2008). In addition, [^18^F] FDG (or ^3^H- or ^14^C-labeled glucose analogues) and other tracers have been used as a means to test cell metabolism and viability in a large number of drug development studies. However, the ideal in vitro incubation conditions for the testing of such tracers has not been defined and experiments have often been performed under conditions, where cellular hypoxia may be poorly controlled. For example, in dense cell cultures the actual cellular oxygen tension may be profoundly reduced compared to the oxygen tension in the equilibrating gas due to slow oxygen diffusion and high cellular respiration (so-called peri-cellular hypoxia). The confounding influence may be particular problematic when experiments are performed under low (but not zero) oxygen tensions. For example, under brief episodes of hypoxia mitochondrial respiration may be maintained down to very low oxygen levels with a half maximum respiration at 0.2–0-3 mmHg in isolated mitochondria and ~ 1 mmHg in intact cells (Steinlechner-Maran et al. 1996). Thus, when doing experiments at low O_2_ levels, even minor differences between equilibration gasses and intracellular O_2_ caused by cellular respiration may profoundly influence energy metabolism. Diffusion-limitations may similarly per se affect the retention of tracers (and drugs in treatment experiments), which of course is of general importance and not only a concern for tracers that are tested for hypoxia-specificity. To what extent tracer diffusion-limitations are problematic, depends on tracer uptake capacity (e.g., slow transmembrane diffusion versus rapid active transport) and the size of the tracer, since large molecules will diffuse more slowly. Incubation under gentle orbital shaking conditions may effectively offset the problems with insufficient oxygen and tracer delivery, but the great majority of experiments are still conducted under non-shaking conditions. Low oxygen tensions protects against radio-induced DNA damage and during anoxic conditions radioresistance is typically 3-fold enhanced compared to well-oxygenated conditions when experiments are performed in glass Petri dishes (Sorensen et al. 2013; Gray et al. 1953). The radioprotective effect of anoxic incubation is much reduced in plastic Petri dishes and this finding has been attributed to the presence of significant amounts of oxygen in plastic (e.g., polystyrene) that may be released during incubation, especially for experiments with a rather short duration of the gas equilibrium period (Gray et al. 1953; Chapman et al. 1970; Chapman et al. 1969). Nonetheless, several studies that have characterized the hypoxia-selectivity of tracers or hypoxia-activated/hypoxia-selective cytotoxins (e.g., tirapazamine, TH-302) have used plastic Petri dishes or well plates (Kumar et al. 2018; Sun et al. 2015). The primary purpose of this study was to define the possible confounding influence of applying non-shaking conditions and traditional cell culturing plasticware. We focused on quantification of cellular retention of hypoxia-sensitive radiotracers since their uptake may be particularly sensitive to a poorly controlled microenvironment caused by cellular oxygen consumption and/or releasable oxygen in the substrata as well as diffusive barriers.

## Materials and methods

The following cell lines were tested: SiHa (squamous cell carcinoma, SCC, of the cervix), FaDu_DD_ (Head and Neck SCC), SW948 (colon adenocarcinoma, AC) and the mammary AC’s MCF7 and MDAMB231. SiHa was cultured in MEM whereas remaining cell lines were cultured in HEPES-buffered DMEM. Medium was supplemented with 10% FCS, non-essential amino acids, pyruvate and penicillin/streptomycin. For experiments, cells were seeded in 5 cm Polystyrene or in Borosilicate glass dishes and grown until confluent (except SW948, which do not form dish-covering monolayers but rather tends to grow in clusters). For the testing of scaling-down the number of cells, while maintaining a high degree of confluence, cells were seeded as 5 discrete droplets of cells in 75 μl medium in Polystyrene Petri dishes. Following attachment of cells (> 6 h), dishes were flooded with medium and cells were grown until defined droplet areas were confluent. The chemical properties of Borosilicate glass Petri dishes makes seeding in discrete droplets challenging. Instead, 75 μl cell suspension were seeded on 9 mm circular glass cover slips in compartmentalized (but with free fluid movement between compartments) glass Petri dishes with four compartments each holding one cover slip. Following attachment of cells (> 6 h), dishes were flooded with medium and cells were grown until confluent.

Since the key aim of this study was to single out the influence of substrata and shaking conditions on tracer retention, experiments were performed using standard culturing media to avoid any induction of forced or adaptive metabolic responses (other than those induced by the hypoxic challenge), that may compromise the study conclusiveness. In accordance, when ready for experiments, cells were supplied with 7.5 ml pre-warmed fresh MEM (SiHa) or DMEM (other cell lines) medium and transported to the PET department and incubated without dish lids in gas tight chambers from Billups-Rothenberg, modified so that tracers can be added through small sealable holes while maintaining a defined gas atmosphere. Typically, experiments were done using gentle orbital shaking during the entire pre-equilibration and tracer retention periods. Following a 2 h pre-equilibration period, 20 μl tracer, equivalent to ~ 0.5 MBq for [^18^F] FDG and ~ 1 MBq for [^18^F] FAZA, were added to each dish through the sealable holes using a Hamilton syringe. For SiHa and FaDu_dd_, complementary experiments were also performed without shaking during the tracer retention period. In these experiments, cells were shaken for 30 min initially to ensure initial gas equilibration followed by a 90 min period without shaking. Immediately after tracer addition, cells where briefly shaken for 5 min to ensure appropriate mixing of tracer and medium. Cells were pre-incubated with 21% O_2_, 0.5% O_2_ or 0% O_2_ (all with 5% CO_2_ and N_2_ as balance) delivered from gas flasks using a flow of 5 l/min for 30 min followed by 0.3 l/min for the duration of the experiment. To reduce evaporation, gas mixtures were humidified using glass gas washing bottles, which resulted in a total fluid loss by evaporation of less than 0.5% (unpublished observations, M Busk).

Following a tracer retention period of 1 h ([^18^F]FDG) or 3 h ([^18^F]FAZA), cells were harvested. In short, a 100 μl medium sample, used for normalization, was drawn from each dish and transferred to counting vials. Afterwards, remaining medium was discarded and cells were rinsed thoroughly in saline by rapid submersion in three large beakers each containing 5 l of isosmotic NaCl. After wash, cells were collected in 0.75 ml of water using cell scrapers and transferred to counting vials. Each dish was then carefully rinsed with an additional 0.75 ml of water, which was transferred to their respective vials to ensure removal of any residual cell material. All 4 coverslips from a given dish were washed and transferred to a single vial. Finally, radioactivity was determined using a Packard well counter.

### Calculations and statistics

All measurements were decay corrected and subsequently normalized to the activity concentration in the same dish to compensate for any minor unintentional dish-to-dish variability in tracer, which improves accuracy/reproducibility compared to assuming that activity concentration is identical in all dishes (unpublished observations, Busk M). Finally, cellular tracer retention was expressed relative to control cells (21% O_2_). The influence of plating material and shaking conditions were compared for a given cell line, plating approach (droplets/confluent dishes) and oxygenation level using a paired two-tailed T-test. The level of significance was *P* < 0.05 and data are presented as means ± standard error of mean (SEM).

## Results

**Fig. 1:**
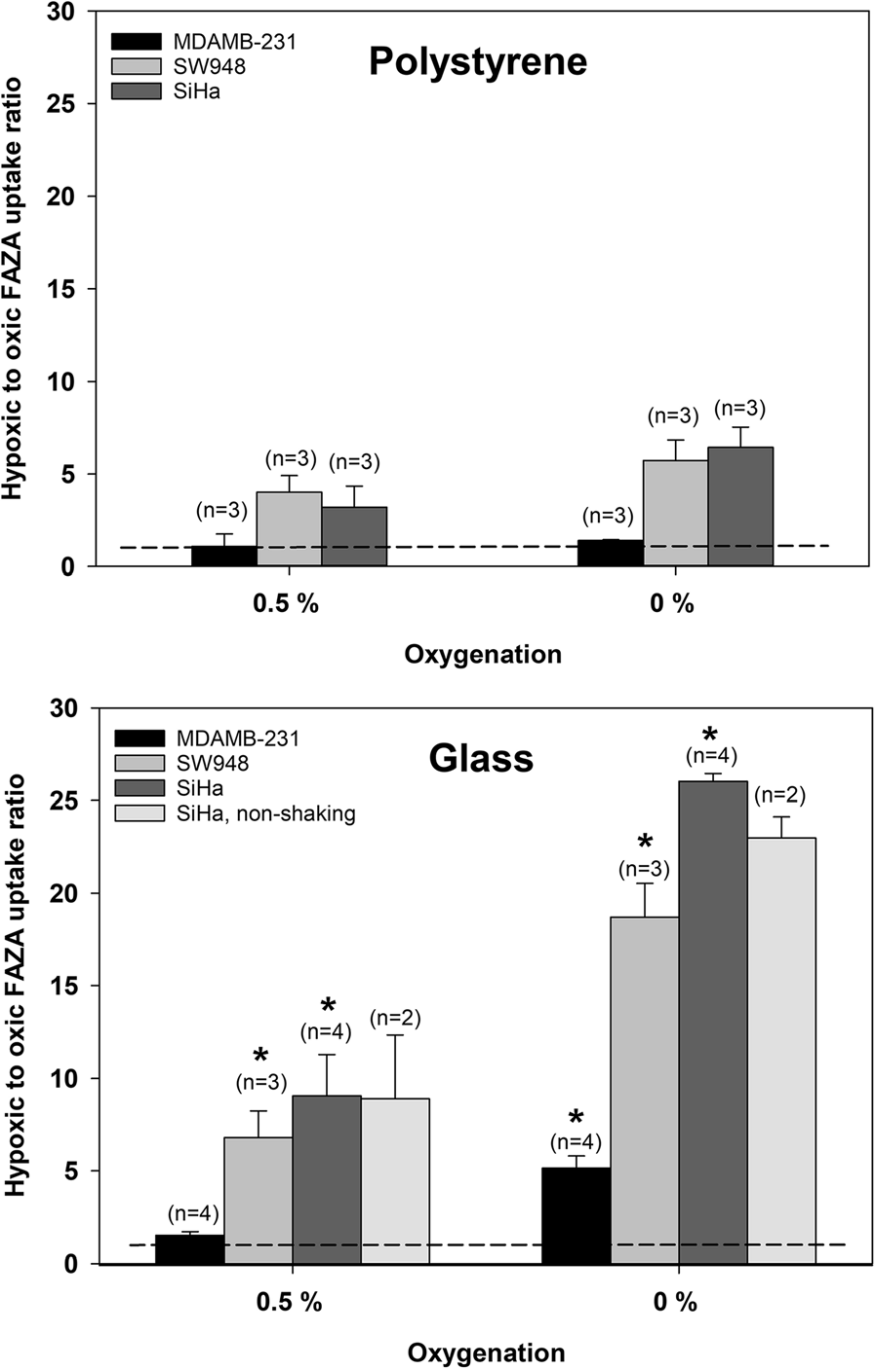
Oxygenation-dependent [^18^F] FAZA binding and its dependency on plating material. [^18^F] FAZA uptake under hypoxic (0.5% O_2_) and anoxic (0% O_2_) gas equilibration is expressed relative to the radioactivity measured in well-oxygenated (21% O_2_) cells. The dotted line represents a ratio of 1, that is, no stimulation of uptake. Values are means (± SEM). The number of independent experiments for each condition is given in parentheses. The asterisks denotes a significant difference between glass and polystyrene at a given oxygenation level (*p* < 0.05)

**Fig. 2:**
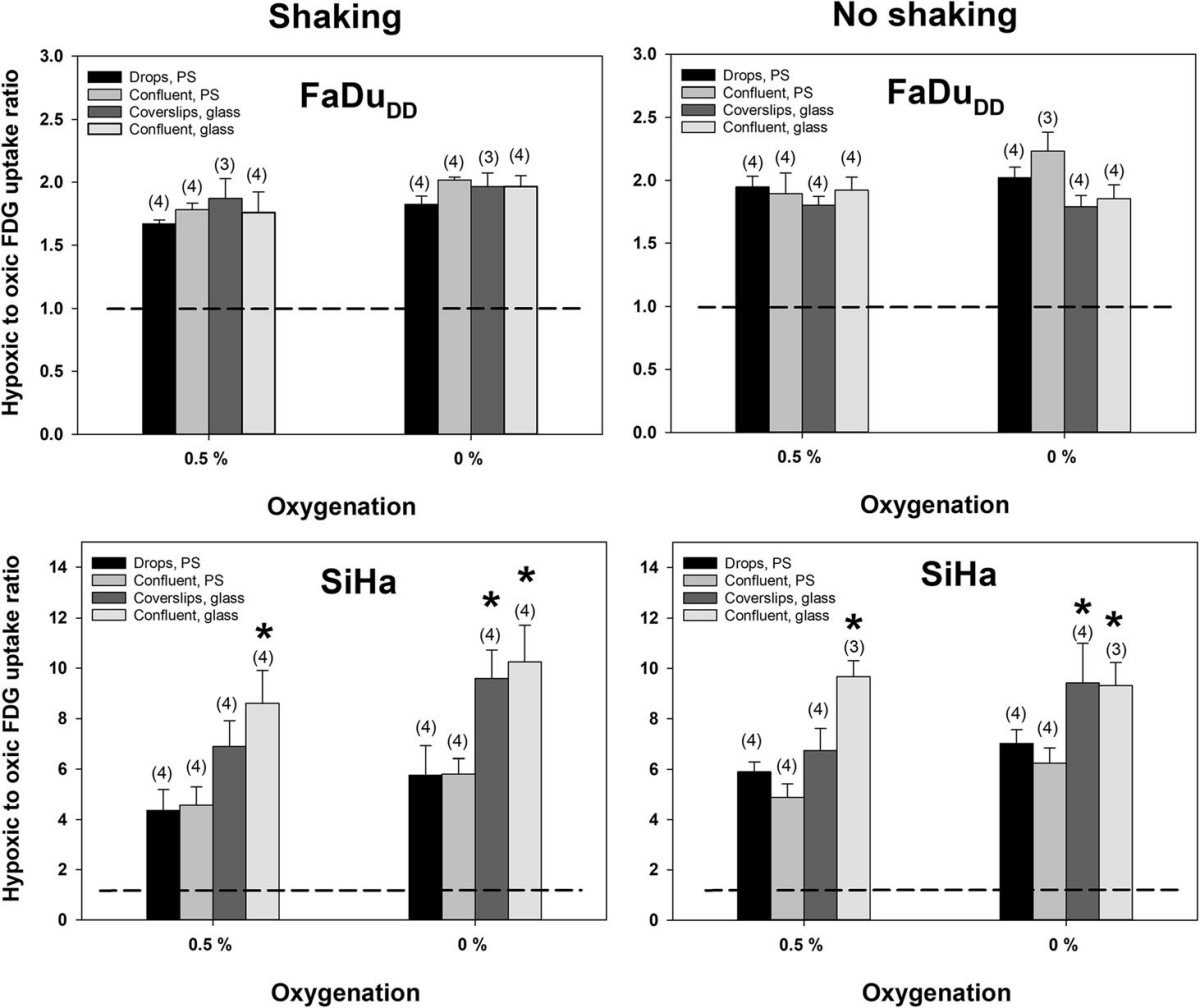
Oxygenation-dependent [^18^F] FDG uptake and its dependency on plating material, shaking conditions and cell number (growth area). [^18^F] FDG retention under hypoxic (0.5% O_2_) and anoxic (0% O_2_) gas equilibration is expressed relative to the radioactivity measured in well-oxygenated (21% O_2_) cells. Values are means (± SEM). The number of independent experiments for each condition is given in parentheses. The asterisks denotes a significant difference between glass and polystyrene at a given oxygenation, cell number (growth area) and shaking condition (*p* < 0.05)

**Fig. 3:**
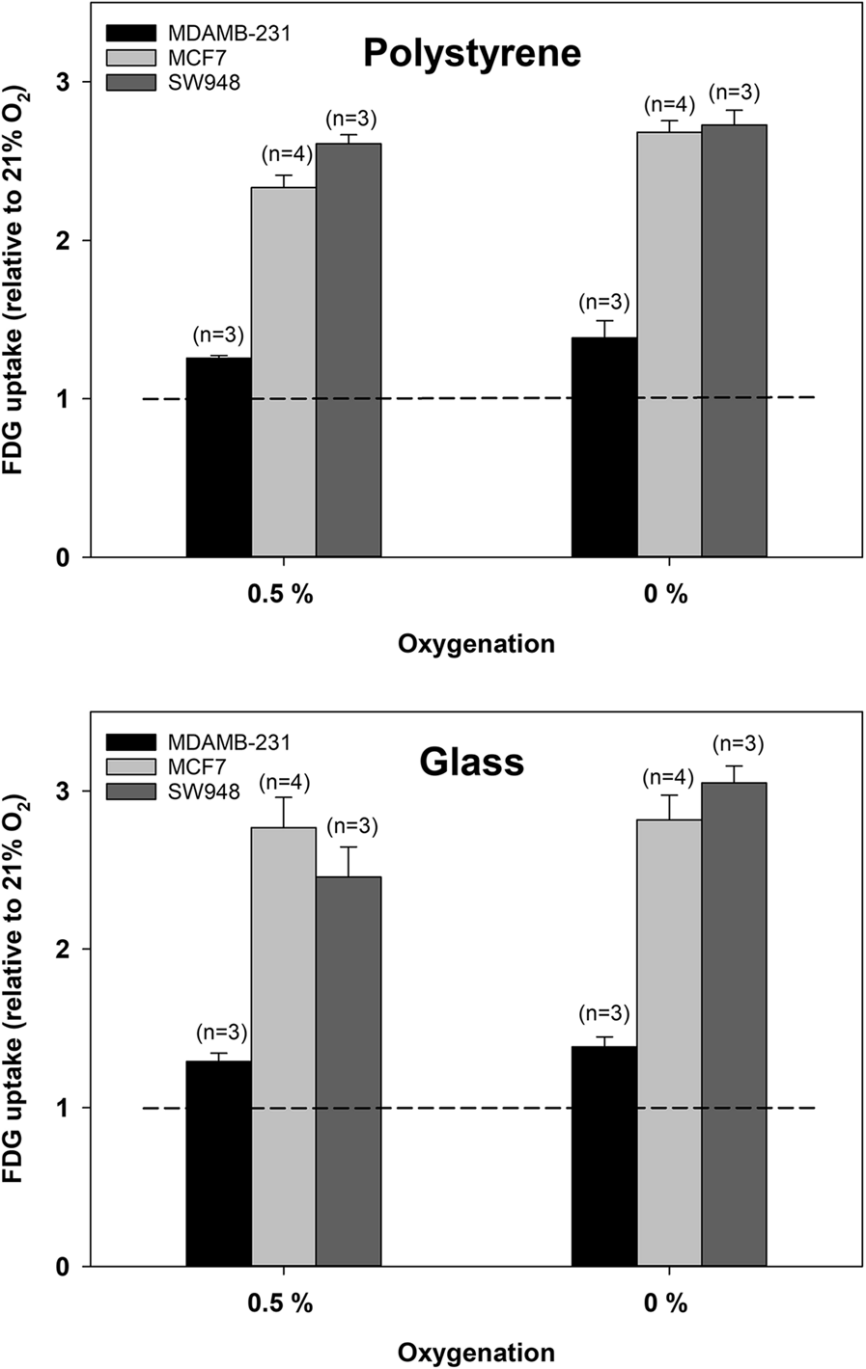
Oxygenation-dependent [^18^F] FDG uptake and its dependency on plating material using orbital shaking in a further panel of cell lines. Values are means (± SEM). The number of independent experiments for each condition is given in parentheses

Total cellular tracer content at harvest was cell line dependent but was consistently below 2% for [^18^F] FAZA and 1% for [^18^F] FDG in confluent Petri dishes suggesting that tracer availability is buffered sufficiently for the entire labeling period. Scaling down cell number, by reducing the effective growth area, further reduced retention by 3–6 fold. Figure 1 shows the oxygen dependence of cellular [^18^F] FAZA uptake in confluent cells, expressed as ratios between hypoxic and well-oxygenated cells following a 3 h tracer-loading period. Low oxygen levels stimulated [^18^F] FAZA retention profoundly, but with distinct intra-cell line differences. Of note, uptake was much lower in cells grown on polystyrene than on glass, and stimulation under 0.5% O_2_ on glass was similar or even higher than uptake in cells in polystyrene dishes equilibrated to an anoxic gas. We also assessed the influence of plating material on the oxygenation-dependent stimulation of glycolysis (from [^18^F] FDG retention) in a cell line panel (Figs. 2 and 3). For FaDu_DD_ and SiHa cells we extended the experimental design to also include a comparison between stagnant and shaking conditions, as well as the reliability of downscaling of cell number by reducing the effective growth area (Fig. 2). The hypoxia-induced stimulation of [^18^F] FDG retention varied widely between cell lines, ranging from a barely detectable response in MDAMB-231 (Fig. 3) to a 10 fold increase under anoxia in SiHa cells (Fig. 2) grown on glass. The influence of plating material was less pronounced for [^18^F] FDG than for [^18^F] FAZA, but polystyrene suppressed the stimulatory effect of hypoxia in SiHa cells significantly. Reducing the cell-number-to-medium volume, while maintaining confluence, by downscaling of the effective growth area, was robust and did not systematically affect hypoxic-to-normoxic tissue ratios nor inter-experiment variability (Fig. 2).

## Discussion

Our results showed that plating material influence the hypoxia-driven retention of [^18^F] FAZA profoundly in all cell lines, whereas a suppressive effect of polystyrene on [^18^F] FDG uptake only was observed in SiHa cells. Of note, [^18^F] FAZA retention data suggests that the average effective cellular oxygen pressure experienced during the tracer loading period is above 0.5% O_2_ in polystyrene attached cells even after a 2 h pre-equilibration period under shaking conditions. This equals a pO_2_ of ~ 3.5 mmHg assuming a normal pressure of 760 mmHg and a water vapor pressure of 47 mmHg when using a gas humidifier. Besides posing a problem for the development of hypoxia-selective tracers, such differences may influence any experiment where exact control of oxygen levels are critical, including studies on hypoxia-induced gene expression and hypoxia activated prodrugs. Our results do not decisively rule out other explanations for reduced anoxia-driven tracer uptake in polystyrene grown cells, but our observations corroborate with seminal studies showing that radiosensitivity during an anoxic gas challenge is elevated when using polystyrene and that it relates to the presence of substantial and releasable quantities of O_2_ in plastics (Chapman et al. 1970; Chapman et al. 1969). Other types of plastic such as permanox may reduce, but not fully eliminate, the problem (Zeman et al. 1990). To avoid unwanted influx of oxygen from the substrate, pre-equilibrated plastic-ware has been applied in some studies on bioreductive cytotoxins and the kinetics of hypoxia-induced genes (Jamieson et al. 2018). However, such an approach requires a hypoxia work-station and more importantly, prevents seeding and growing of cells under identical standard culturing conditions prior to experimentation and gas challenge, which in turn may induce unknown biological differences and experimental bias that cannot be resolved unequivocally. The confounding influence of plastic-ware in hypoxia research have generally gained footing in radiotherapy research, but is less appreciated outside this field. In most cell lines, [^18^F] FDG uptake was uninfluenced by plating material, suggesting that glycolysis is fully activated at pO_2_ levels higher than those required for significant reduction of [^18^F]FAZA. This may appear somewhat surprising since the partial pressure at which respiration is reduced to 50% of the maximal rate at saturating oxygen is reported to be around or below 1 mmHg in intact cells (Steinlechner-Maran et al. 1996). However, during more prolonged hypoxia (hours) adaptive changes typically cause respiration to drop, and glycolysis to increase, at much higher values (so-called oxygen conformance) with a P_50_ in the range of 5–15 mmHg. In accordance, a 6 h exposure to relatively mild hypoxia of 1.5% O_2_ (~ 10 mmHg) stimulated FDG retention in 2 tumor cell lines (Minn et al. 1996). Interestingly, in SiHa cells, which unlike typical tumor cells have a largely non-glycolytic phenotype under aerobic conditions, and thus experience larger adaptive glycolytic flux changes when mitochondrial ATP synthesis ceases, a significant difference was observed between polystyrene and glass. Whether this relates to true cell-to-cell line differences in pO_2_ threshold values that affects respiration due to difference in oxygen conformance behavior (see above) or rather reflects increased assay sensitivity in the highly responsive SiHa cell line is unclear, but highlights that also in studies on hypoxia-induced changes in energy metabolism the choice of culturing material may influence results. Indeed, in such studies the use of well-plates is common and this may further exaggerate the problem of prolonged oxygen release and also result in differences between centrally and peripherally located wells, so-called edge effects. Of note, in a hypothetical study conducted in polystyrene dishes, one would erroneously conclude that [^18^F] FDG and [^18^F] FAZA performs equally well as markers of severe hypoxia in SiHa cells (compare Figs. 1 and 2) and that [^18^F] FAZA displays no hypoxia-selectivity in MDAMB-231 cells (Fig. 1).

Another, often underappreciated, problem is that cells alter their local and global environment by consumption of tracers, O_2_, metabolites and possibly even drugs, which may lead to hypoxia and insufficient diffusive delivery of tracers and drugs. During long-term experiments in dense cultures alterations in medium composition (e.g., glucose) may also be a concern (see also next paragraph). For example, (Pettersen et al. 2005) showed that pericellular oxygen tension in confluent stagnant cultures may deviate substantially from pO_2_ in equilibration gasses, and concluded that this may affect any study that correlates cell biology to oxygen levels. One approach to optimize control of the pericellular microenvironment, is the use of freshly trypsinized cells maintained in shaken suspension in glass vials. This procedure has been valuable in several settings including the testing of hypoxia PET tracers (Minn et al. 2000; Minn et al. 1996), but may be less useful for delicate metabolic and gene expression studies, since several studies have shown that detached cells are stressed, and may behave differently than attached cells (Danhier et al. 2013; Ren et al. 2004). Therefore, when possible the use of attached cells are ideal. Not all cells attach firmly to glass, but coating may effectively overcome these problems. A simple, but not commonly used, way to overcome or limit diffusion barriers is the use of gentle orbital shaking, since convection will ensure fast and effective gas equilibration of medium and reduce the effective thickness of still layers. This may be particularly relevant when studying low, but non-zero, levels of oxygenation, where the relative influence by cell self-consumption may lead to large relative changes in oxygenation. Surprisingly, our results showed that [^18^F] FDG retention was unaffected by shaking conditions even at an O_2_ level of 0.5% in dense cultures. A similar result was obtained for [^18^F] FAZA retention in SiHa cells grown on glass (Fig. 1). These results suggests that even in experiments where proper control of pO_2_ is required non-shaking conditions may be appropriate at least when using the experimental conditions in our study. Still orbital shaking may be necessary in other settings including very dense cultures or when testing tracers/drugs that diffuses more slowly (e.g., higher MW).

A key advantage of using tracers such as [^18^F] FDG, ^11^C-acetate and ^11^C-methionine to assess cellular metabolism, is that measurements can be performed rapidly under reasonably stable culturing conditions. In contrast, traditional enzymatic assays determines uptake rates based on concentration changes in metabolites in medium samples, which by necessity prevents measurements of fluxes at stable medium and cell number conditions. A second problem is that detectable changes in medium composition may require long incubation periods, which leads to substantial evaporation even when using humidified gasses. In our acute experiments, the total cellular tracer retention was typically below 2% of total added dose (quantified in medium samples prior to cell harvest), suggesting that medium-volume-to-cell content was sufficient to ensure near stable conditions. Nonetheless, in many settings, such as studies on cell metabolism under chronic tumor-microenvironment-mimicking conditions with nutritional deprivation, further reducing the cell number may be advantageous or even required to dampen inappropriate changes in medium metabolites that affects tracer retention by competition (e.g., glucose, acetate) or indirectly by changing cellular metabolic state (e.g., lactic acidosis). However, very low cell numbers may compromise accuracy due to random variations in the radioactive disintegration rate and background. This may partly be circumvented by prolonging the post-harvest radioactivity measurement time or by increasing the added dose, but the latter strategy may not be acceptable due to radioactivity concerns or costs (for long-lived tracers). In accordance, we also assessed the reliability of downscaling the number of cells, without adjusting measurement time and added dose, while maintaining a high degree of confluence, by growing cells in discrete areas in polystyrene dishes or on glass cover slips. Downscaling typically resulted in an acceptable total radioactivity of 2000–3000 CPM under oxic conditions. In addition, the hypoxia induced stimulation of ^18^F] FDG was in agreement with results obtained when growing cells as dense cultures in whole dishes. Whether this is true in other settings and for other tracers with lower uptake (such as [^18^F] FAZA under oxic conditions) must be determined before a given application. An added advantage when using cover slips is that cell washing and radioactivity assessment is very easy to perform since meticulous cell scraping and cell suspension collection is not required, and that cells do not, or only slowly, spread to the Petri dish bottom. The coverslip approach is thus particular useful when doing prolonged incubations where strictly defined environmental conditions are required at a high degree of confluence. In cells on cover slips, SiHa typically retained around 0.25% of added [^18^F] FDG under anoxia in 1 h in MEM (≈5 mM glucose). Assuming that the relative affinity for [^18^F] FDG retention and glucose use (the so-called “lumped constant”) is one (Barrio et al. 2019) and that glucose metabolic rate is maintained under hypoglycemic conditions, then a 24 h anoxic pre-incubation of SiHa cells at 1 mM glucose would lower medium glucose by approximately 30%. This quantity can be further reduced to ~ 7.5%, when only using a single cover slip, suggesting that by appropriate downscaling such experiments are feasible.

## Conclusions

We have demonstrated that choosing an appropriate plating material may be crucial for obtaining reliable results in studies involving hypoxia, especially in studies involving brief (hours) exposures to near-anoxic conditions. Surprisingly, we obtained similar results under stagnant and forced convection conditions. Robustness was not compromised by down scaling, which may be required in prolonged studies where careful control of the medium composition is required. Finally, it must be stressed that as with any bioassay testing and development, our results may not be generalizable to different settings in other laboratories using other cell types, culture densities and tracers.

## Data Availability

All data generated or analysed during this study are included in this published article.

## Abbreviations

[^18^F]FDG: ^18^F-fluoro-2-deoxyglucose
[^18^F]FAZA: ^18^F-fluoroazomycin-arabinoside
PET: Positron emission tomography
SPECT: Single photon emission computed tomography
SCC: Squamous cell carcinoma
AC: Adenocarcinoma
MEM: Minimum Essential Medium Eagle
DMEM: Dulbecco’s Modified Eagle’s Medium
HEPES: 4-(2-Hydroxyethyl)piperazine-1-ethanesulfonic acid
